# Optoelectronic Nanodevices

**DOI:** 10.3390/nano10030520

**Published:** 2020-03-13

**Authors:** Minas M. Stylianakis

**Affiliations:** Department of Electrical & Computer Engineering, Hellenic Mediterranean University (HMU), Estavromenos, 71410 Heraklion, Greece; stylianakis@hmu.gr; Tel.: +30-2810-379775

Over the last decade, novel materials such as graphene derivatives, transition metal dichalcogenides (TMDs), other two-dimensional (2D) layered materials, perovskites, as well as metal oxides and other metal nanostructures have centralized the interest of the scientific community [1,2,3,4]. Due to their extraordinary physical, optical, thermal, and electrical properties, which are correlated with their 2D ultrathin atomic layer structure, large interlayer distance, ease of functionalization, as well as bandgap tunability, these nanomaterials have been applied towards the development or the improvement of innovative optoelectronic applications, as well as the expansion of theoretical studies and simulations in very fast-growing fields of energy (photovoltaics, energy storage, fuel cells, hydrogen storage, catalysis, etc.), electronics, photonics, spintronics and sensing devices [5,6,7,8]. The continuous nanostructure-based applications development provides the confidence to significantly improve existing products and to explore the design of materials and devices with novel functionalities [9,10,11].

This Special Issue reports some of the most recent trends and advances in the interdisciplinary field of optoelectronics, including 24 original research articles and two review papers. Most articles focus on light emitting diodes (LEDs) and solar cells (SCs), including organic, inorganic and hybrid configurations, while the rest concern some excellent studies on photodetectors, transistors, as well as on other well-known dynamic optoelectronic devices, as depicted in Figure 1. In this context, this exceptional collection of articles is directed at a broad scientific audience of chemists, materials scientists, physicists, and engineers, with the goals of not only pointing out the potential of innovative optoelectronic applications incorporating nanostructures but also inspiring their realization.

The first section of the special issue includes five original articles from the broad field of solar cells technology. First, the article by Deng at al. [22], reports on the preparation of a Ti porous film supported NiCo_2_S_4_ nanotube and its role as a fluorine doped tin oxide (FTO)/Pt alternative counter electrode (CE) in CdS/CdSe quantum-dot-sensitized solar cells (QDSSCs). The novel porous counter electrode, prepared by acid etching and a two-step hydrothermal method, exhibited better electrocatalytic properties, improved loading, as well as higher stability, resulting in a photovoltaic performance improvement of the fabricated QDSSC by 240%, compared to the conventional FTO/Pt based one. Next, Ho et al. [23] demonstrate their efforts to boost the performance and the output power of textured silicon solar cells through plasmonic forward scattering. They individually inserted a single and a double layer of two-dimensional indium NPs within a SiN*_x_*/SiO_2_ double-layer antireflective coating. As a result, the conversion efficiency, for both approaches, was improved compared to the reference cell up to 5%. They also investigated the effect of In NPs layers within the double layer of ARC on light-trapping performance of the devices at different slopes. Finally, in a same manner, the electrical output power was evaluated, exhibiting an enhancement over 50%.

In another study, Shi et al. [24] present the growth of high performance ultrathin MoO_3_/Ag transparent electrodes via thermal evaporation at different deposition rates, by assessing their optical, electrical, and morphological characteristics. They proved that the synergy of MoO_3_ with silver, even as a very thin nucleation layer, was beneficial for the fabrication of uniform, semitransparent, and highly conductive porous films. The said ultrathin electrode was used in inverted organic solar cells as top semitransparent electrode, exhibiting a significant PV performance (2.76%) when the device was illuminated from the top side. The article by Svrcek et al. [19] deals with the development of low roughness InN thin films through the pulsed metalorganic vapor-phase epitaxy (MOVPE) process. The optimized InN/p-GaN photoelectric heterojunction exhibited excellent electron extraction efficiency compared to the literature for similar studies based on pulsed MOVPE.

The ternary configuration has shown a great potential in the field of organic solar cells. In this context, Stylianakis et al. [16] synthesized a novel graphene derivative with favorable energy levels to the binary blend materials of the active layer of a typical inverted organic solar cell. The new graphene-based molecule was incorporated in ink form as the third component within the active layer, in various ratios. In that way, highly efficient inverted ternary organic solar cells (OSC) devices were realized, exhibiting a performance improvement by 13%, compared to the control device, leading to a record PCE value of 8.71%. Power conversion efficiency (PCE) enhancement was attributed to the cascade effect, facilitating electron transport from the active layer to ITO bottom electrode, as well as the morphology improvement between the interfaces of the binary blend components. The last article of this section is by Chen et al. [25], who examined the effect of solvent polarity, as well as the concentration of the precursor methylammonium iodide (MAI) on the morphology of MAPbI_3_ perovskite thin films. They found that better coverage and compactness were achieved according to the longer alkyl chain of the solvent (lower polarity), although the film’s roughness was higher. Upon the parameters’ optimization, highly efficient perovskite solar cells were prepared with a champion PCE of 16.66%.

The second section consists of ten original contributions concerning the field of LEDs. Feng et al. [12] present their study on the combination of localized surface plasmon (LSP) and quantum wells (QWs), deriving from the incorporation of silver NPs into the holes of a photonic crystal within the *p*-GaN layer of a green LED. In order to demarcate the impact of light and e-beam induced excitations on LSP–QW coupling mechanism, they adopted a 3D finite difference time domain (FDTD) numerical simulation model during the photophysics measurements, while they suggest a beneficial way towards the energy dissipation reduction in Ag NPs. Next, Yan et al. [13] evaluated the performance of green and red CdSe quantum dots (QDs) based LED devices, at various excitation wavelengths, by assessing the photoluminescence (PL) of the respective thin films. In addition, the indefinite role of a V-pits-embedded InGaN/GaN superlattice (SL) in green LEDs performance improvement was explored by Liu et al. [18], who achieved to improve the device’s external quantum efficiency (EQE) by ~30%, upon the development of the V-pits-embedded InGaN/GaN SL layer. They used scanning electron microscopy (SEM) synergistically to a room temperature cathodoluminescence (CL) to validate light emission properties of InGaN/GaN multiple quantum wells (MQWs), proving that V-pits may act as a barrier for carriers’ diffusion into nonrecombination centers. Moreover, Chen et al. [21] prepared highly fluorescent and uniform MAPbBr_3_ thin films by tuning the content of octylammonium bromide (OAB) additive within the perovskite precursors. In this manner, high performance perovskite-based LEDs were fabricated, exhibiting very high luminance and luminous current efficiency values, due to high exciton binding energy of the nanocrystals grain size, resulting in nonradiative recombination mitigation, as well as emission efficiency augmentation.

Li et al. [26] report on their efforts towards the improvement of color-conversion efficiency (CCE) and stability of quantum-dot-based light-emitting diodes upon the incorporation of a blue anti-transmission film (BATF). They proved that under the optimum tradeoff between the BATF thickness and QDs concentration, both CCE and luminous efficacy can be significantly improved by over 42% and 24%, respectively. The study by Zhou et al. [27] proposes the design of two novel p-type layers a) a gradually reduced indium content p-InGaN and b) a *p*-GaN. The first one acted as an ideal replacement of *p*-AlGaN, while the second boosted the light output power of a GaN-based green LED. Indeed, the champion green LED device exhibited an improvement by ~14% compared to the reference one in light intensity while the indium content was gradually reduced from 10% to 0%, as confirmed by experimental and simulation studies. In another theoretical report on GaN-based LEDs, Jin et al. [28] state an error-grating simulation model and recommend some nano-grating strategies, including the use of alternative materials, as well as the adaption of different fabrication structural parameters towards the improvement of light extraction efficiency of LEDs. They compare the results from the literature regarding the use of several materials as patterned bottom reflection layer and suggest that the incorporation of SiO_2_ nanorod array (NR) is the optimum approach.

The fabrication of a multilayered transparent conductive electrode (TCE) for AlGaN-based UV LEDs, with improved optoelectronic properties, of the structure ITO/Ga_2_O_3_/Ag/Ga_2_O_3_ is suggested by Wang et al. [29]. In that way, the complex TCE exhibited very high transmittance values at 365 nm and extremely low specific contact resistance, upon annealing. The combination of two single ITO layers is demonstrated by Zhao et al. [15] for GaN-based UV LEDs, in order to improve light extraction efficiency (LEE) and to reach low-resistance ohmic contact with the layer of p-GaN. The concept was implemented using laser direct writing and was further supported by numerical simulations. In the last article of the section, Tang et al. [30] describe their great efforts on LEE enhancement in flip-chip (mini) GaN-based LEDs, through the management of the total internal reflection at sapphire/air interface, using a tetramethylammonium hydroxide (TMAH) based etching. In this manner, hierarchical prism-structured sidewalls were formed in the device, to boost light output power, due to light trapping, and thus scattering phenomena exploitation.

Three novel studies and a review article in the field of photodetectors are presented in the third section of this SI. More specifically, Shih et al. [31] fabricated p- and n-type Si heterojunction photodetectors incorporating graphene oxide (GO) of different oxidation degrees. They proved that the oxidation degree, adjusted by tuning the total of hydrogen peroxide during the oxidation process, increased the photoresponse in case of *n*-type Si heterojunction devices and vice versa in case of *p*-type ones. In addition, Xue et al. [17] demonstrate their dual approach regarding a) the controllable synthesis of high quality 2D perovskite platelets, compatible to conventional substrates with a melting point over 100 °C and b) the following fabrication of high-performance photodetectors. On the other hand, Lee et al. [20] report on the preparation of an ultrahigh sensitivity CdTe microdots-based photodetector onto a bottom bismuth coated ITO/glass substrate. The devices exhibited excellent durability and efficiency under stress conditions mainly due to piezo-phototronic effect, which highly affected the height of the Schottky barrier. A review article, by Shi et al. [32] is the last contribution of the section, summarizing the current insights and achievements in the field of organic photomultiplication photodetectors, due to trap assisted carrier tunneling effect, while alternative operational mechanisms, future perspectives, and challenges are also considered.

Novel contributions related to other dynamic devices are also included in the fourth section, which consists of three research articles and a review one. Guo et al. [33] successfully tuned the geometry of GaN-based nanobricks to be incorporated as efficient dielectric metasurface towards the manipulation of orthogonal linear polarizations simultaneously in the visible light. Moreover, they conceived and constructed a polarization beam splitter (PBS), as well as the required focusing lenses at the wavelength of 530 nm, featuring the great options of visible light optical devices. In another simulation study, Ma et al. [34] suggest a metamaterial structure composed of a gold split-ring and a graphene one, in order to reinforce the electromagnetically induced transparency (EIT) effect in the mid infrared (MIR) region and to create a tunable transparency window, by adjusting the coupling distance and/or the Fermi level of the graphene-based split-ring. Their results could be exploited towards the development of various light management nanodevices. Wu et al. [35] demonstrate the development of a reusable and flexible tunable filter through an advanced electrowetting fluid-manipulation technology. They observed that upon the simulated adjustment of the period of the grating structure filter, the reflection of CMY (cyan, magenta, yellow) primary colors was accomplished. On the other hand, the significantly shorter device’s response time was attributed in the fast speed of electrowetting fluid manipulation process. The section closes with an extensive summary regarding the most recent accomplishments in the field of dynamically tunable metasurface-based applications incorporating liquid crystals (LCs) by Ma et al. [36]. They provide various rational architectures, as well as the most common factors to adjust the optical properties of LCs. In this context, they list various combinations between LCs and a range of metasurfaces that could be accomplished, aiming to increase their compatibility with high performance functional dynamic nanodevices. 

The last part of the Special Issue incorporates original articles demonstrating the realization of other efficient optoelectronic nanodevices, such as field emission (FE) devices and transistors, respectively. First, the article by Stylianakis et al. [14] compares the FE performance of four solution-processed approaches including the use of polymeric and fullerene derivatives’ composites towards the development of rGO-based in different ratios cold cathodes for FE devices. The prepared devices displayed excellent stability, higher field enhancement factor, and much lower turn-on field compared to the reference n+-Si/rGO FE devices. Celebrano et al. [37] close the Special Issue demonstrating their investigation on the photocurrent behavior of an erbium-doped and co-doped with oxygen silicon-based transistor at room temperature (RT), upon alteration of the laser source wavelength. They proved that the photocurrent strongly depends both on the power, as well as the frequency of the laser source, while the potential incorporation of Er-doped silicon in RT single photon resonators is highlighted.

To sum up, the present Special Issue entitled “Optoelectronic Nanodevices” assembles original research contributions from various subfields of optoelectronics. This collection showcases outstanding experimental and simulation studies pointing out the potential of novel organic, inorganic, hybrid composites, as well as 2D nanomaterials to be incorporated in order to enhance the performance and to extend the lifetime of conventional optoelectronic applications.

## Figures and Tables

**Figure 1:**
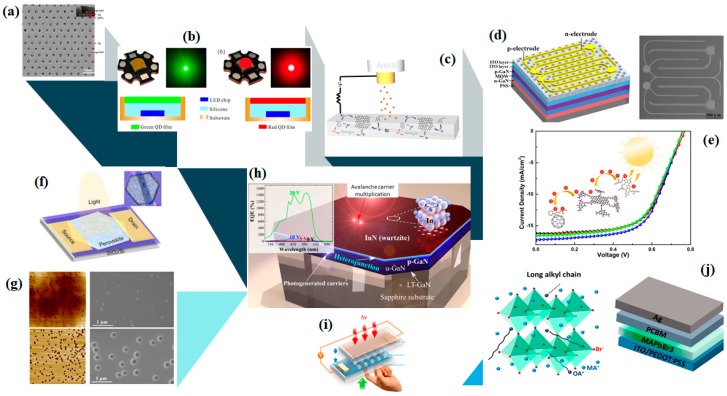
Various configurations of the optoelectronic devices reported in the Special Issue. (**a**) Ag–photonic crystal (PhC) SEM image. Reproduced with permission from [12]; (**b**) real representations and schematic diagrams of a green and a red quantum-dot light emitting diode (LED). Reproduced with permission from [13]; (**c**) representation of a field emitter cold cathode based on graphene oxide r(GO):organic charge transfer materials composites. Reproduced with permission from [14]; (**d**) schematic representation of a patterned double-layer indium tin oxide (ITO) ultraviolet (UV) LED. Reproduced with permission from [15]; (**e**) typical *J-V* curves of a ternary organic solar cells (OSC) device and schematic representation of charge transfer between the active layer materials. Reproduced with permission from [16]; (**f**) schematic representation of a two-dimensional (2D) perovskite platelet phototransistor. Reproduced with permission from [17]; (**g**) atomic force microscopy (AFM) and SEM images of green LEDs with and without InGaN/GaN superlattice. Reproduced with permission from [18]; (**h**) schematic representation of an InN/p-GaN photoelectric heterojunction. Reproduced with permission from [19]; (**i**) a CdTe microdots array photodetector configuration. Reproduced with permission from [20]; (**j**) configuration of a light emitting diode (PeLED) based on octylammonium substituted perovskite. Reproduced with permission from [21]. Copyright of all figures MDPI publisher, 2020.
